# Docosahexaenoic Acid-Acylated Astaxanthin Esters Exhibit Superior Renal Protective Effect to Recombination of Astaxanthin with DHA via Alleviating Oxidative Stress Coupled with Apoptosis in Vancomycin-Treated Mice with Nephrotoxicity

**DOI:** 10.3390/md19090499

**Published:** 2021-08-31

**Authors:** Hao-Hao Shi, Ying Guo, Li-Pin Chen, Cheng-Cheng Wang, Qing-Rong Huang, Chang-Hu Xue, Yu-Ming Wang, Tian-Tian Zhang

**Affiliations:** 1 College of Food Science and Engineering, Ocean University of China, Qingdao 266003, China; shh@stu.ouc.edu.cn (H.-H.S.); guoying@stu.ouc.edu.cn (Y.G.); chenlipin@stu.ouc.edu.cn (L.-P.C.); wangchengcheng@stu.ouc.edu.cn (C.-C.W.); wangyuming@ouc.edu.cn (Y.-M.W.); 2 Department of Food Science, The State University of New Jersey, Rutgers, 65 Dudley Road, New Brunswick, NJ 08901, USA; qhuang@sebs.rutgers.edu; 3 Laboratory for Marine Drugs and Bioproducts, Pilot National Laboratory for Marine Science and Technology (Qingdao), Qingdao 266237, China

**Keywords:** nephrotoxicity, astaxanthin, DHA, monoesters, diesters

## Abstract

Prevention of acute kidney injury caused by drugs is still a clinical problem to be solved urgently. Astaxanthin (AST) and docosahexaenoic acid (DHA) are important marine-derived active ingredients, and they are reported to exhibit renal protective activity. It is noteworthy that the existing forms of AST in nature are mainly fatty acid-acylated AST monoesters and diesters, as well as unesterified AST, in which DHA is an esterified fatty acid. However, no reports focus on the different bioactivities of unesterified AST, monoesters and diesters, as well as the recombination of DHA and unesterified AST on nephrotoxicity. In the present study, vancomycin-treated mice were used to evaluate the effects of DHA-acylated AST monoesters, DHA-acylated AST diesters, unesterified AST, and the recombination of AST and DHA in alleviating nephrotoxicity by determining serum biochemical index, histopathological changes, and the enzyme activity related to oxidative stress. Results found that the intervention of DHA-acylated AST diesters significantly ameliorated kidney dysfunction by decreasing the levels of urea nitrogen and creatinine, alleviating pathological damage and oxidative stress compared to AST monoester, unesterified AST, and the recombination of AST and DHA. Further studies revealed that dietary DHA-acylated AST esters could inhibit the activation of the caspase cascade and MAPKs signaling pathway, and reduce the levels of pro-inflammatory cytokines. These findings indicated that the administration of DHA-acylated AST esters could alleviate vancomycin-induced nephrotoxicity, which represented a potentially novel candidate or therapeutic adjuvant for alleviating acute kidney injury.

## 1. Introduction

Acute kidney injury (AKI) is characterized by the rapid decline of renal function and the accumulation of metabolic waste [1,2]. Drug-induced renal damage has become a global public health concern. Vancomycin, as a glycopeptide antibiotic, is usually used to treat many types of bacteria; however, it exhibits serious side effects, especially nephrotoxicity [3,4]. Current treatments for AKI only provide symptomatic relief in the development of kidney disease rather than complete cure. Therefore, it is still an urgent and unfulfilled mission to find a suitable approach to alleviating nephrotoxicity. Although the underlying mechanism of nephrotoxicity remains extremely complicated, the development of natural active substances with effective nephroprotection has attracted researchers’ attention [2,5].

**Figure 1:**
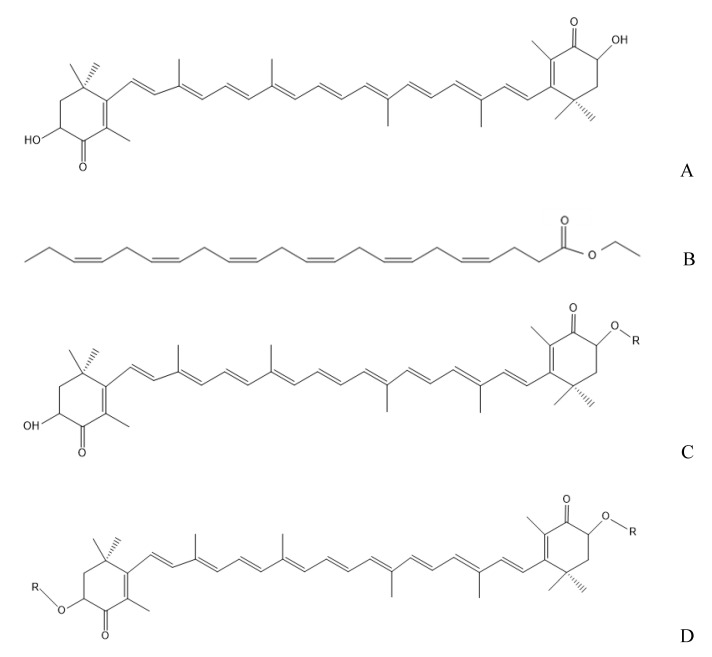
The structure of the astaxanthin (**A**), docosahexaenoic acid in the form of ethyl ester (DHA, **B**), DHA-acylated astaxanthin monoester (**C**) and diester (**D**). R indicates DHA.

Astaxanthin (AST, Figure 1A) is one of the most important carotenoid pigments naturally existing in seafood, such as crabs and shrimps. Accumulating research projects have focused on the healthy benefits of AST, including anti-inflammatory, immunomodulatory, anti-cancer, and neuroprotective effects [6,7]. Moreover, recent studies have shown that AST could protect against renal injury induced by ischemia-reperfusion [8]. It is noteworthy that the existing forms of AST in nature are mainly free states and fatty acid-acylated AST esters. It has been reported that there were 70% diesters, 12% monoesters, and 10% unesterified AST in red crab [9]. AST esters are mainly composed of AST and several different fatty acids, such as palmitic acid, oleic acid, linoleic acid, docosahexaenoic acid (DHA, Figure 1B), and eicosapentaenoic acid (EPA). Recent studies showed that AST esters had better biological activities, including anti-cancer activity, liver protection, and antioxidant function compared with unesterified AST [10]. A great number of animal and clinical data have shown that DHA possessed a significant alleviation on kidney disease [11,12]. Our previous research showed that dietary supplementation with DHA-acylated AST diester could alleviate the behavioral deficits in the mice with Parkinson’s disease and cognitive disorders in model mice of Alzheimer’s disease, which was better than that of unesterified AST [13,14]. However, no reports focus on the different bioactivities of diesters, monoesters, and unesterified AST, especially kidney injury. Moreover, DHA is chemically unstable and susceptible to oxidation. The recombination of DHA with AST may be a good strategy to increase their stability and synergistic bioactivities. Nevertheless, it is still unclear whether the recombination of DHA and unesterified AST achieve equivalent physiological activity with DHA-acylated AST esters in alleviating nephrotoxicity.

Therefore, the present research aimed to evaluate the different effects of DHA-acylated AST monoesters (Figure 1C), DHA-acylated AST diesters (Figure 1D), unesterified AST, and the recombination of AST and DHA in alleviating vancomycin-induced nephrotoxicity by determining serum biochemical index, histopathological changes, and the enzyme activity related to oxidative stress. Meanwhile, the possible underlying molecular mechanism was investigated by Western blotting analysis.

## 2. Results

### 2.1. Effects on Vancomycin-Induced Kidney Dysfunction

**Figure 2:**
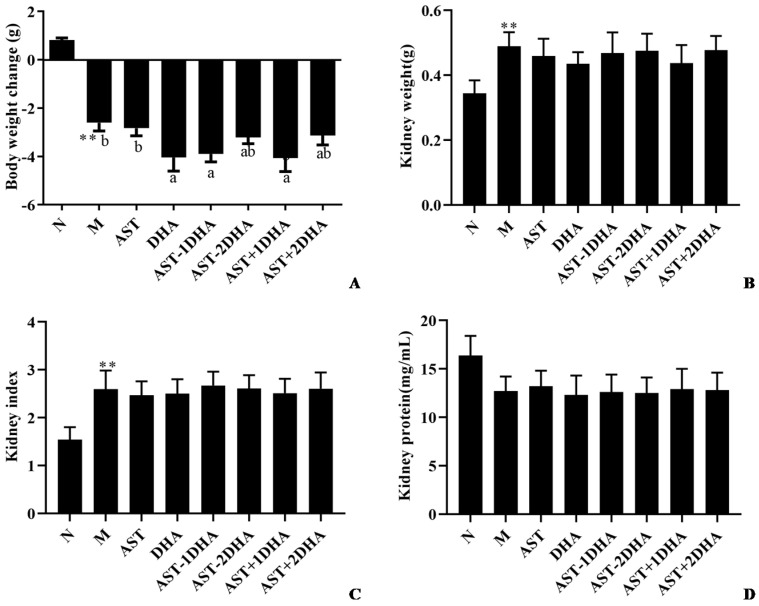
Effects of different samples on body weight change (**A**), kidney weight (**B**), kidney index (**C**), and kidney protein concentration (**D**) in mice. All data were expressed as mean ± S.E.M. for 8 mice. ** Significantly different compared to normal group (*p* < 0.01). Different letters represent significant differences among experimental groups.

After intraperitoneal injection with vancomycin, the body weight was dropped sharply (14.7%, *p* < 0.01), and the kidney weight and index were significantly increased (42.2% and 68.5%, *p* < 0.01) compared with the normal group (Figure 2). No significant differences for kidney weight and index and kidney protein concentration were observed among all vancomycin-treated groups (*p* > 0.05).

**Figure 3:**
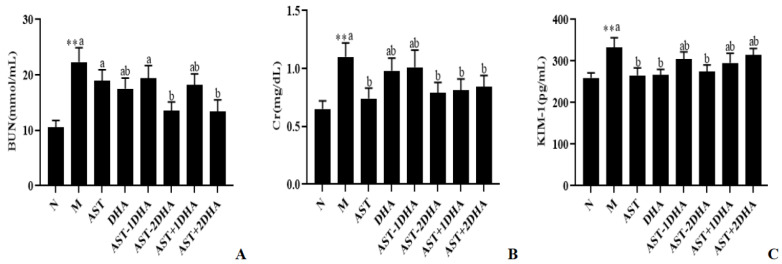
Effects of different samples on the serum BUN (**A**), Cr (**B**), and KIM-1 (**C**) in mice. All data were expressed as mean ± S.E.M. for 8 mice. ** Significantly different compared to normal group (*p* < 0.01). Different letters represent significant differences among experimental groups.

BUN, Cr, and KIM-1 were associated with the renal glomerular filtration rate that was widely used in clinical diagnosis. As shown in Figure 3, kidney function biomarkers (BUN, Cr, and KIM-1) levels were strongly raised after the injection of the vancomycin-treated group (110%, 69.2%, and 28.2%, *p* < 0.01), suggesting that a successful acute kidney injury model was successfully established. Compared with the model group, only pretreatment with AST-2DHA and AST+2DHA significantly suppressed the BUN levels by 39.4% and 40.0%, respectively, which was very close to the normal level. Moreover, AST-2DHA, AST+1DHA, AST+2DHA, and AST could clearly reduce the level of Cr (*p* < 0.05) in serum compared to the model group, while no obvious changes were found in the DHA and AST-1DHA groups (*p* > 0.05). Furthermore, AST-2DHA showed significant advantages in decreasing the level of KIM-1 compared with the model group (20.4%, *p* < 0.05), which exhibited similar effects in comparison with AST and DHA. Unfortunately, no significant effects were observed in AST-1DHA and recombination groups in decreasing the level of KIM-1 (*p* > 0.05). On the whole, dietary administration with AST-2DHA could significantly inhibit vancomycin-induced abnormal increase in biochemical parameters related to renal function.

Next, we analyzed the level of DHA in the kidney of mice (Table 1). The content of DHA in the kidney was clearly reduced in the model group compared with the normal group (69.3%, *p* < 0.01). Dietary AST-2DHA could remarkably elevate the DHA level compared with the model group (84.9%, *p* < 0.05), which was better than AST-DHA and the recombination of AST and DHA.

### 2.2. Effects of Different Samples on Vancomycin-Induced Renal Pathological Change

**Figure 4:**
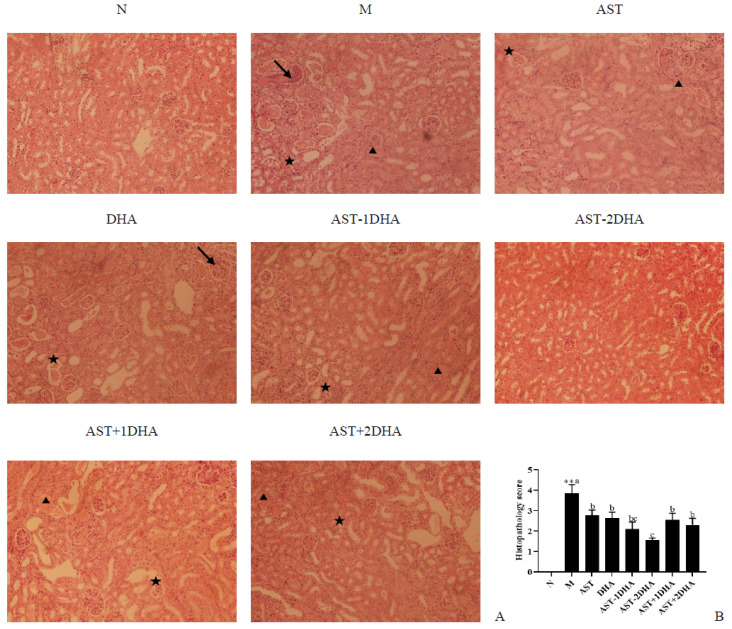
Histopathological results of vancomycin-induced nephrotoxicity as assessed by H&E staining of kidney tissues (**A**) (100× magnification) and results of total histopathological scores reflecting tubular damage in each group (**B**). Star indicates glomerular necrosis; the triangle indicates vacuolization, and the arrow indicates inflammatory infiltration. All data were expressed as mean ± S.E.M. for 8 mice. ** Significantly different compared to normal group (*p* < 0.01). Different letters represent significant differences among experimental groups.

Following the injection of vancomycin, severe changes, including distortion of overall renal morphology, dilation and necrosis of renal tubules, and interstitial inflammation, were found in the kidney (Figure 4). The intervention of different substances could alleviate renal pathological damage in varying degrees. Remarkably, DHA-acylated AST esters, especially AST-2DHA, could remarkably ameliorate vancomycin-induced renal pathological changes in comparison with the recombination of AST and DHA (*p* < 0.05).

### 2.3. Effects on Vancomycin-Induced Activation of The Mitochondrial Apoptosis Pathway

**Figure 5:**
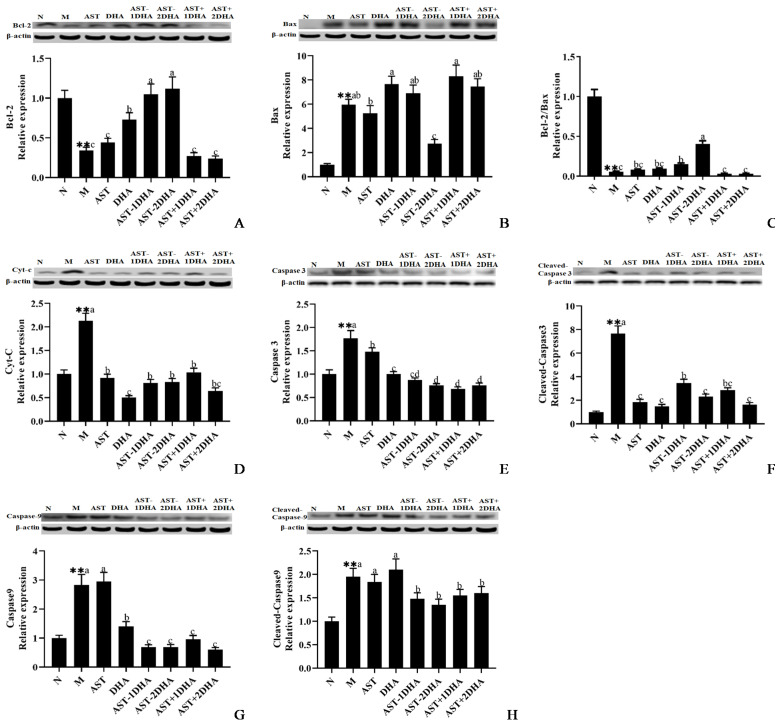
Expressions of Bcl-2, Bax, Bcl-2/Bax, Cyt-C, Caspase 3, Cleaved-Caspase 3, Caspase 9, and Cleaved-Caspase 9 in the kidney were analyzed by Western blotting with β-actin as the loading control of total proteins (**A**–**H**). The band density was quantified by scanning densitometry. All data were expressed as mean ± S.E.M. for 8 mice. ** Significantly different compared to normal group (*p* < 0.01). Different letters represent significant differences among experimental groups.

The mitochondrial pathways of apoptosis have been considered to be critical in the development of acute kidney injury [15]. The protein expression of pro-apoptotic Bax and Cyt-C was markedly elevated, and the protein expression of anti-apoptotic Bcl-2 was significantly inhibited (66.1%, *p* < 0.01) in the model group (Figure 5) compared with that of the normal group. The supplementation with AST-2DHA and AST-1DHA strongly increased the expression of Bcl-2 and Bcl-2/Bax ratio compared with the model group (*p* < 0.05). DHA also markedly elevated the Bcl-2 expression (*p* < 0.05). However, there was no significant difference in the recombination of DHA and AST groups in comparison with the model group (*p* > 0.05). Expectedly, the administration of AST-2DHA significantly suppressed the protein expression of Bax and Cyt-C compared with the model group (53.9% and 60.9%, *p* < 0.05), while other intervened substances could only inhibit the protein expression of Cyt-C rather than Bax.

The injection of vancomycin significantly activated the protein expression of Caspase 3, cleaved-Caspase 3, Caspase 9, and cleaved-Caspase 9 (Figure 5). Specifically, the administration of AST-2DHA significantly inhibited vancomycin-induced cleavage of Caspase 3, which was better than AST-1DHA. AST+2DHA strongly decreased the protein expression of cleaved-Caspase 3 compared with AST+1DHA. Interestingly, the intervention of AST and DHA also markedly decreased the protein expression of cleaved-Caspase 3 (*p* < 0.05). Similarly, increased Caspase 9 and cleaved-Caspase 9 protein expression in the model group was attenuated after treatment with different substances in varying degrees (*p* < 0.05). Notably, DHA-acylated AST esters and recombination groups exhibited a similar effect in inhibiting the cleavage of Caspase 9.

### 2.4. Effects on Vancomycin-Induced Activation of MAPKs Signal Pathway

**Figure 6:**
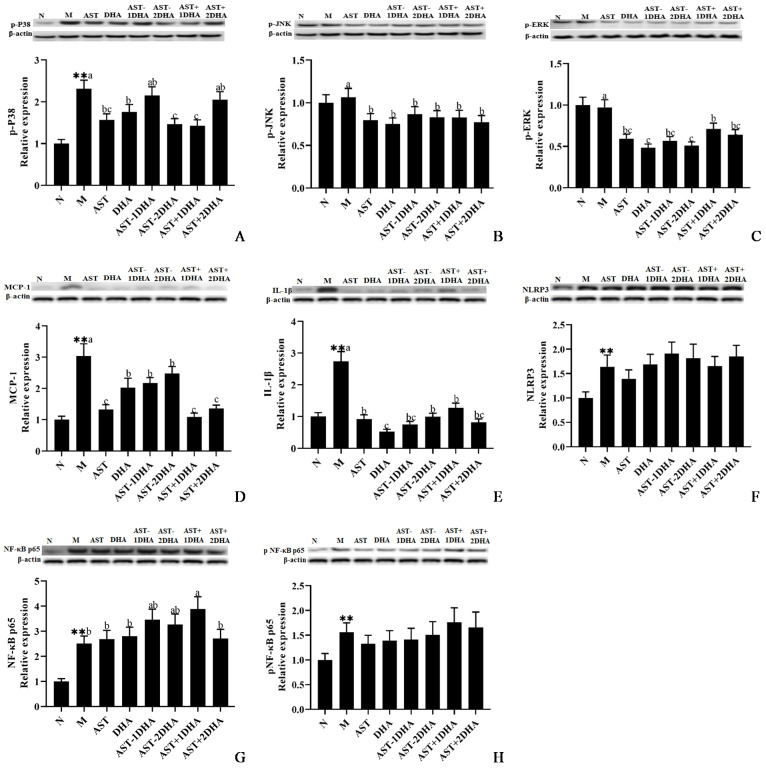
Expressions of p-P38, p-JNK, p-ERK, IL-1β, MCP-1, NLRP3, NF-κB p65, and p-NF-κB p65 in the kidney were analyzed by Western blotting with β-actin as the loading control of total proteins (**A**–**H**). The band density was quantified by scanning densitometry. All data were expressed as mean ± S.E.M. for 8 mice. ** Significantly different compared to normal group (*p* < 0.01). Different letters represent significant differences among experimental groups.

MAPKs signal pathway was regarded to be closely revolved in the process of nephrotoxicity [16], Compared with the normal group, vancomycin treatment could strikingly increase the protein expression of phosphorylated P38 (*p* < 0.01, Figure 6A). AST-2DHA had a more potent effect on suppressing the protein expression of p-P38 among all vancomycin-treated groups (*p* < 0.05), which was similar to AST+1DHA. Furthermore, supplementation of DHA and AST also clearly inhibited the expression of p-P38 compared with the model group (*p* < 0.05). However, there were no significant differences among AST-1DHA, AST+2DHA, and model groups in the protein expression of p-P38 (*p* > 0.05). The protein expression of p-JNK in the AST-2DHA group was markedly decreased by about 23.6% compared with the model group (*p* < 0.05), and there were no significant differences among all substance-treated groups (*p* > 0.05, Figure 6B). Moreover, AST-2DHA and DHA had a potent effect on suppressing the protein expression of p-ERK among all vancomycin-treated groups (*p* < 0.05, Figure 6C). Besides, administration with other active substances also clearly suppressed the expression of p-ERK compared to the model group (*p* < 0.05).

### 2.5. Effects on Vancomycin-Induced Inflammatory Pathway

It is well known that the inflammatory pathway was closely related to the development of kidney disease [5]. The protein expression levels of inflammatory cytokines, including the pro-inflammatory cytokines IL-1β and MCP-1, were clearly raised after the injection of vancomycin compared with that of the normal group (Figure 6D,E). The administration of AST-2DHA markedly reduced the expression level of IL-1β by 63.8% compared with the model group (*p* < 0.05), and there was no statistical difference in the protein expressions between AST-1DHA and AST-2DHA group (*p* > 0.05). In addition, the expression of IL-1β in the recombination groups was less than that in the model group (*p* < 0.05). It was noteworthy that supplementation with DHA had a more potent effect on suppressing the protein expression of IL-1β among all vancomycin-treated groups (*p* < 0.05). Moreover, the intervention of AST-2DHA remarkably inhibited the protein expression of MCP-1 compared with the model group (*p* < 0.05). Dietary supplementation with AST-DHA and DHA also had similar effects on reducing the protein expression of MCP-1. Interestingly, the AST alone administration and the recombination of DHA and AST were more effective than AST-2DHA on inhibiting the protein expression of MCP-1 (*p* < 0.05).

The secretion of IL-1β and MCP-1 was elevated by the activation of the NF-κB signaling pathway. NF-κB was a well-known pro-inflammatory transcription factor. Vancomycin remarkably increased the protein expression level of NLRP3 compared with the normal group (64.0%, *p* < 0.01). However, there were no significant changes in the protein expression of NLRP3 among all vancomycin-treated groups (*p* > 0.05). Likewise, both the protein level of NF-κB p65 and p-NF-κB p65 were clearly elevated in the model group compared with that of the normal group (*p* < 0.01 for both), while the expression of NF-κB p65 and p-NF-κB p65 proteins was not obviously restrained among different substance-treated groups compared with the vancomycin group (*p* > 0.05).

### 2.6. Effects on Vancomycin-Induced Oxidative Stress

**Figure 7:**
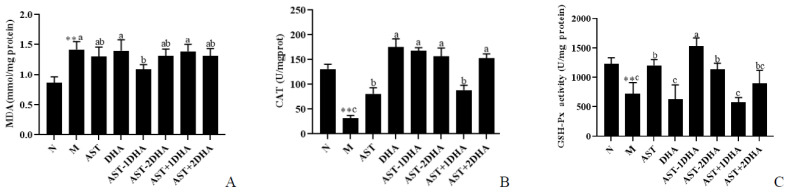
Effects of different samples on MDA, CAT, and GSH-Px of kidney in the mice (**A**–**C**). All data were expressed as mean ± S.E.M. for 8 mice. ** Significantly different compared to normal group (*p* < 0.01). Different letters represent significant differences among experimental groups.

Oxidative stress is a major risk factor that induces mitochondrial dysfunction [17]. Figure 7 showed the effect of different substances on vancomycin-induced oxidative stress in the kidney. It was observed that vancomycin treatment remarkably elevated the level of MDA by 65.1%, as well as reducing the activities of CAT and GSH-Px in the kidney compared with normal mice (75.8% and 41.1%, *p* < 0.01). Compared with the model group, only AST-1DHA significantly suppressed the MDA content in the kidney (23.2%, *p* < 0.05). The administration of different substances, specifically the AST-2DHA, could markedly elevate the CAT enzyme activity in response to varying degrees (*p* < 0.05). Interestingly, the intervention of AST-1DHA, AST+2DHA, and DHA had similar effects. Similarly, the activity of GSH-Px in the AST-2DHA group was significantly higher than the model group (*p* < 0.05), which was similar to that of the AST group. Notably, the effect of AST-1DHA was better than AST-2DHA in enhancing the GSH-Px activity. Meanwhile, there were no significant differences in the GSH-Px activity among the AST+2DHA, AST+1DHA, DHA, and model groups (*p* > 0.05).

## 3. Discussion

At present, an enormous amount of research has shown that nutritional intervention was a feasible and novel strategy for the treatment and alleviation of kidney disease [18]. A great number of studies indicated that AST and DHA possessed adequate protection against kidney disease. Our results showed that dietary supplementation with DHA-acylated AST esters had a significant amelioration on vancomycin-induced acute kidney injury compared with AST or DHA alone administration. Similarly, our previous studies have verified that AST esters significantly improved learning and memory abilities compared with unesterified AST in APP/PS1 transgenic mice [13]. Moreover, the recombination of unesterified AST and DHA was also an effective way for alleviating vancomycin induced-nephrotoxicity, although its effect was less than that of the AST esters. Likewise, our previous studies have found that DHA-acylated AST ester exhibited better effects on alleviating symptoms of Parkinson’s disease than the recombination of unesterified AST and DHA in an animal model induced by 1-methyl-4-phenyl-1,2,3,6-tetra-hydropyridine (MPTP) [14]. We estimate that the different protective effects of DHA-acylated AST esters might be related to their digestion and absorption process in the body. The level of DHA and EPA was significantly reduced in the kidneys of mice with chronic kidney disease [19]. Our previous study has indicated that the absorption capacity of DHA in the AST-2DHA group was higher than that in the AST-DHA [20], and has shown the high level of DHA in the kidney of mice after administration with AST-2DHA, which might be attributed to the significant effect of AST-2DHA on ameliorating nephrotoxicity. In addition, although the absorption capacity of AST was higher in the AST-DHA than in the AST-2DHA in our previous study, the AST content in the small intestinal content in the AST-2DHA group was significantly higher than that in the AST-1DHA group [20], suggesting that there might be an interaction between AST-2DHA and gut microbes. Recently, it has been reported that there was a close relationship between gut microbes and the development of acute kidney injury [21]. We speculated that the gut–kidney axis played an important role in the alleviation of nephrotoxicity for AST-2DHA intervention, which needs to be further studied.

The mitochondrial apoptosis pathway played an increasingly great role in drug-induced nephrotoxicity [22]. An large number of studies have shown that vancomycin treatment could increase the expression of pro-apoptotic proteins (Bax, Cyt-C, Caspase 3, and Caspase 9) and decrease the expression of the Bcl-2 protein [12]. Our results suggested that the administration of DHA-acylated AST diesters was more effective than the recombination of unesterified AST and DHA in increasing the expression of Bcl-2 protein and inhibiting the expression of several apoptotic proteins, especially the changes of Bax and cleaved-Caspase 3. It has also been reported that supplementation of DHA could markedly decrease the protein expression of Cyt-C, Caspase 3, and Caspase 9 and increase the protein expression of Bcl-2 in the brain of mice with MPTP-induced cytotoxicity [23]. Pretreatment of DHA for 2 weeks increased the Bcl-2 protein expression, decreased the Bax expression, and prevented tubular cell apoptosis in mice with kidney ischemia/reperfusion injury [24]. Likewise, our previous study showed that dietary DHA-acylated AST diesters significantly attenuated cognitive disorders via decreasing the protein expression of Caspase 9, Caspase 3, and cleaved-Caspase 3 in the brain of mice with Alzheimer’s disease compared with unesterified AST [13]. However, dietary DHA-acylated AST diesters had no corresponding improvement in regulating Bcl-2 and Bax protein expression in the brain [13]. Moreover, dietary supplementation of AST esters significantly suppressed the protein expression of Bax, Cyt-C, and Caspase 3, as well as upregulated Bcl-2 levels in the brain of mice with Parkinson’s disease [14].

Intensive research demonstrated that the activation of the MAPKs signaling pathway might affect the development of acute kidney injury [25]. A wave of recent research projects has pointed out the abnormal phosphorylation of JNK, ERK, and P38 proteins in the kidney of AKI mice [26]. All of the present results implicated that AST diesters could inhibit the phosphorylation level of P38, JNK, and ERK proteins. Our previous studies have shown that dietary supplementation of DHA significantly inhibited the phosphorylation of JNK and P38 in kidneys of mice with nephrotoxicity as well as in the brain of mice with Alzheimer’s disease or Parkinson’s disease [12,23,27]. Moreover, pretreatment of AST attenuated hepatic ischemia-reperfusion via inhibiting the MAPKs pathway in mice [28]. Furthermore, dietary AST esters clearly restrained the ratio of phosphorylated-JNK/JNK and phosphorylated-P38/P38 while non-esterified AST and the recombination of AST with DHA had no corresponding effect on the MPTP-induced mice with Parkinson’s disease [14].

Emerging evidence indicates that both MAPKs and NF-κB inflammatory pathway together participate in kidney disease. Some inflammatory cytokines and chemokines were released from the damaged kidney tissue to stimulate the activation and infiltration of inflammatory cells when kidney tissue was damaged [5]. The results of this study showed that AST esters, as well as the recombination DHA and AST, could significantly suppress the expression of pro-inflammatory factors (IL-1β and MCP-1) compared with alone administration with DHA or AST, particularly the recombination of DHA and AST on inhibiting MCP-1 expression. Current evidence from experimental animal studies reported that dietary DHA could obviously alleviate liver damage by decreasing the IL-1β level in serum and liver of high-fat diet-treated mice [29]. Moreover, dietary AST for 8 weeks could protect against LPS-induced inflammatory response by inhibiting the activation of NF-κB and MAPKs signaling pathways [30]. In the present study, it was puzzling that supplements of different samples had little effect on the protein expression of NF-κB p65, p-NF-κB p65, and NLRP3 in AKI mice. Our previous study also suggested that the intervention of AST esters could significantly reduce the expression of TNF-α in the brain of APP/PS1 double-transgenic mice to improve cognitive and memory function compared with the unesterified AST; similarly, there was no statistical difference in the expression of NLRP3 among all groups [13]. The difference in efficacy between AST esters and AST might be attributed to the different experimental backgrounds or dosage and duration of intervention.

A large number of studies have shown that oxidative stress was closely associated with drug-induced AKI [22]. Treatment of vancomycin might lead to excessive free radical production, oxidative damage, and lipid peroxidation in the kidney tissue [12,31]. The present results showed that the enzyme activity of CAT and GSH-Px were significantly reduced in the kidney tissue of vancomycin-treated mice. At the same time, the dietary intervention of AST esters could significantly inhibit oxidative stress by decreasing the level of MDA and enhancing the enzyme activity of CAT and GSH-Px in the kidney. Some research projects suggested that dietary supplementation of fish oil could increase the activity of GSH-Px and reduce the level of MDA in the model mice with kidney disease [24]. Likewise, administration with AST exerted a dose-dependent effect in increasing GSH-Px activity as well as decreasing MDA levels in the kidney of mice after burn injury [32]. Our previous studies demonstrated that dietary supplementation with AST ester could significantly elevate the activity of T-SOD compared with the unesterified AST in the brain of APP/PS1 mice [13].

According to the above results, it was puzzling that dietary intervention of AST-1DHA ameliorated vancomycin-induced apoptotic responses and oxidative stress; however, it had no significant effect on BUN, Cr, and KIM-1 in the serum, which might be attributed to the short intervention time (7 days). Accumulating evidence has demonstrated that renal injurious drugs could lead to abnormal renal hemodynamics, immune inflammation, mitochondrial dysfunction, fibrosis, apoptosis, and oxidative stress in the kidney of mice [2]. The abnormal renal hemodynamics was mainly manifested in the decrease in vascular resistance in the glomerulus, which increased the plasma flow in the nephron, elevated glomerular perfusion, and reduced water and salt reabsorption, resulting in changes in renal blood flow and glomerular filtration rate [33]. The abnormal renal hemodynamics was closely related to the increase in BUN, Cr, and KIM-1 in the serum [34]. We speculate that AST-1DHA supplementation might have no significant effect on improving renal hemodynamics, so it did not clearly reduce the content of biomarkers related to kidney function, which needs to be further studied.

## 4. Materials and methods

### 4.1. Chemicals

AST was purchased from Kingsci Biotechnology.Co., Ltd. (Xi’an, China). Vancomycin was provided by Dalian Meilun Biotech Co., Ltd. (Dalian, China). 4-(Dimethylamino) pyridine (DMAP) and 1-(3-dimethylaminopropyl)-3-ethylcarbodiimide hydrochloride (EDCI) were obtained from Shanghai Medpep Co. (Shanghai, China). DHA was purchased from Zhoushan Xinnuojia Co., Ltd. (Zhoushan, China). Assay kits of creatinine (Cr), blood urea nitrogen (BUN), malondialdehyde (MDA), catalase (CAT), and glutathione peroxidase (GSH-Px) were obtained from Nanjing Jiancheng Bioengineering Institute (Nanjing, China). ELISA kits for kidney injury molecule-1 (KIM-1) were purchased from Wuhan USCN Business Co., Ltd. (Wuhan, China).

### 4.2. Preparation and Determination of DHA-Acylated AST Esters

The DHA-acylated AST esters were prepared according to the published methods [35]. 

Briefly, the initial DHA-acylated AST esters were prepared by esterification reaction from unesterified AST, and DHA under the catalytic reaction of DMAP and EDCI. The impurities of the pretreatment products were removed by a range of cleaning solutions, including hydrochloric acid, carbonate solution, and sodium chloride solutions in sequence. Thereafter, the monoester and diester were separated by silica gel column chromatography. The purity was confirmed by high-performance liquid chromatography with diode array detection (HPLC-DAD) using the corresponding standards as references [35]. Finally, the purities of DHA-acylated AST monoester and diester were determined as 88.1% and 92.6%, respectively. The remaining components might be unesterified astaxanthin and DHA based on the esterification reaction principle [35].

### 4.3. Animals and Treatment

Male BALB/c mice (8-week-old) were reared under an air-conditioned room at a temperature of 23 ± 2 °C, a 12 h/12 h light/dark cycle, and a relative humidity of 62 ± 10%. The experiments were approved by the Laboratory Animal Care Animal Ethics Committee of College of Food Science and Engineering, Ocean University of China (Qingdao, China). Each mouse was housed in a separate cage. Mice were randomly divided into 8 groups (n = 8): normal group (N), model group (M), AST, DHA, DHA-acylated AST monoester (AST-1DHA), DHA-acylated AST diesters (AST-2DHA), recombination of non-esterified AST with equivalent DHA (AST+1DHA), and recombination of non-esterified AST with double the amount of DHA (AST+2DHA) groups. The mice were gavaged with different samples once a day. The AST levels in the AST, AST-1DHA, AST-2DHA, AST+1DHA, and AST+2DHA groups were 100 mg/kg per day. The level of DHA in the DHA group was 100 mg/kg per day based on the human intake level. The mice received vancomycin (400 mg/kg per day) by intraperitoneal injection after pretreatment with different substances for three days. The mice were sacrificed on the 8th day, and the serum and kidney, brain, liver, and muscle tissues were collected. These samples were quickly frozen by liquid nitrogen and stored in a −80 °C refrigerator for future research.

### 4.4. Biochemical Assays

The serum levels of Cr, BUN, and KIM-1 were determined according to the instructions of commercial assay kits. They were prepared to determine the activities of MDA, CAT, T-AOC, GSH, and GSH-Px in 10% tissue homogenate, according to the kit instructions.

### 4.5. Histological Analysis

The kidney tissues fixed in 4% paraformaldehyde were dehydrated and embedded in paraffin, eventually stained by hematoxylin and eosin (H&E). Pathological levels in the kidney were evaluated and graded based on the extent of kidney damage, including tubular necrosis, inflammatory infiltration, interstitial edema, and tubular atrophy or dilatation. Tubular damage degree was evaluated and scored based on the percentage of tubular necrosis. Grade 0: 0–10%, Grade 1: 11–25%, Grade 2: 26–50%, Grade 3: 50–75%, Grade 4: 76–100% tubular damage [36]. Mean scores were calculated by counting 10 different fields for each kidney slide.

### 4.6. Western Blotting Assessment

The kidney tissues were homogenized by Radio Immunoprecipitation Assay (RIPA) protein lysate, and the concentration was determined using an enhanced bicinchoninic acid (BCA) protein assay kit [12]. The Western blotting was analyzed for Bcl-2 (1:1000, Abcam Inc., Cambridge, MA, USA), Bax (1:1000, Abcam Inc., Cambridge, MA, USA), Cyt-C (1:1000, Abcam Inc., Cambridge, MA, USA), Caspase 3 (1:1000, Abcam Inc., Cambridge, MA, USA), cleaved-Caspase 3 (1:1000, CST Inc., Danvers, MA, USA), Caspase 9 (1:1000, Abcam Inc., Cambridge, MA, USA), cleaved-Caspase 9 (1:1000, CST Inc., Danvers, MA, USA), p-P38 (1:1000, CST Inc., Danvers, MA, USA), p-JNK (1:1000, CST Inc., Danvers, MA, USA), p-ERK (1:1000, CST Inc., Danvers, MA, USA), IL-1β (1:1000, CST Inc., Danvers, MA, USA), MCP-1 (1:1000, CST Inc., Danvers, MA, USA), NF-κB p65 (1:1000, CST Inc., Danvers, MA, USA.), p NF-κB p65 (1:1000, CST Inc., Danvers, MA, USA), and NLRP3 (1:1000, CST Inc., Danvers, MA, USA), as previously described [12].

### 4.7. Statistical Analysis

All data were expressed as the mean ± standard error of measurement (S.E.M) and evaluated using Student’s t-test and Tukey’s test. The statistical significance of differences among histopathological scores was tested by the non-parametric Kruskal–Wallis test, followed by the Mann–Whitney U test. A *p* value of <0.05 was considered statistically significant.

## 5. Conclusions

**Figure 8:**
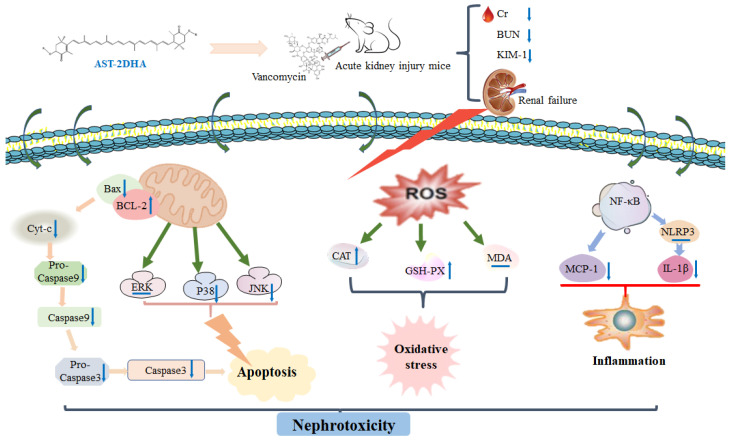
The possible underlying mechanism involved in the alleviation of nephrotoxicity after dietary supplementation with DHA-acylated astaxanthin diester (AST-2DHA).

In conclusion, dietary DHA-acylated AST esters, especially diesters, exhibited a superior choice for beneficial effects on alleviating nephrotoxicity over the recombination of AST and DHA by reducing the levels of BUN and Cr in serum, ameliorating renal histopathological changes and regulating the enzyme activity related to oxidative stress in vancomycin-treated mice. Further studies indicated that DHA-acylated AST esters could suppress apoptosis and MAPKs signaling pathway, as well as downregulating pro-inflammatory levels, as shown in Figure 8. Taken together, the obtained results indicated that DHA-acylated AST esters exhibited the potential and might be used as a therapeutic adjuvant to ameliorate drug-induced nephrotoxicity.

## Figures and Tables

**Table 1:** The level of DHA in the kidney of mice.

(mg/g)	N	M	AST	DHA	AST-1DHA	AST-2DHA	AST+1DHA	AST+2DHA
DHA	3.46 ± 0.41	1.06 ± 0.15c **	1.13 ± 0.09c	1.36 ± 0.11b	1.42 ± 0.13b	1.96 ± 0.21a	1.26 ± 0.09bc	1.76 ± 0.15a

All data were expressed as mean ± S.E.M. for 8 mice. ** Significantly different compared to normal group (*p* < 0.01). Different letters represent significant differences among experimental groups.

## Data Availability

Not applicable.
